# A systematic review of parental burnout and related factors among parents

**DOI:** 10.1186/s12889-024-17829-y

**Published:** 2024-02-05

**Authors:** Xiaohe Ren, Yingying Cai, Jingyi Wang, Ou Chen

**Affiliations:** School of Nursing and Rehabilitation, Cheeloo College of Medicine, Shandong University, Jinan, China

**Keywords:** Parental burnout, Parenting burnout, Factors, Parents, Systematic review

## Abstract

**Background:**

Parenting is both a complex and stressful endeavor, so parents sometimes experience parenting burnout. The main objective of this study was to provide an overview of factors related to general parental burnout (PB) among parents with at least one child based on the Ecological Systems Theory (EST).

**Methods:**

PubMed, Web of Science, EBSCO, CNKI and WanFang were systematically searched for studies published from 2010 to July 2023 for peer-reviewed articles using keywords extracted from Medical Subject Headings such as “parenting”, “parental”, “burnout”, “psychological burnout”, “burn-out syndrome”. Studies were included if they described associations between factors and PB among parents of children aged 0-18 years old in the general population, and published in an English or Chinese language peer-reviewed journal. The Quality Assessment Tool for Studies with Diverse Designs (QATSDD) was employed to assess the risk of bias of included studies.

**Results:**

Of 2037 articles, 26 articles met the inclusion criteria. Based on the Ecological Systems Theory (EST), we found that microsystem-individual factors such as gender, educational level, income, parental personality, internalization of maternal parental motivation, unmitigated communion, self-compassion and concern for others, alexithymia, anxiety and depressive symptoms, parental perfectionism, resilience, low self-esteem and high need for control, mother's attachment style were identified as being associated with parenting burnout. Mesosystem-interpersonal factors involve parent-child relationship and marital satisfaction. The exosystem-organizational or community factors include the number of children in the household, neighborhood and the number of hours spent with children, child's illness, child's behavior problems and social support. The macrosystem-society/policy or culture factors are mainly personal values and cultural values.

**Conclusions:**

This systematic review found several factors that have been investigated in relation to PB. However, the majority of the factors were reported by one or two studies often implementing a cross-sectional design. Nevertheless, we still recommend that health policymakers and administrators relieve parenting burnout among parents with children by adjusting these modifiable factors.

**Supplementary Information:**

The online version contains supplementary material available at 10.1186/s12889-024-17829-y.

## Introduction

The phenomenon of burnout occurs in any activity that elicits frequent and intense stress responses, and parenting is a complex, stressful activity that is highly susceptible to parenting burnout (PB) [1]. The phrase “parental burnout” refers to a set of undesirable symptoms resulting from parental role and long-term parenting stress [2]. Parental burnout progressively becomes a severe social problem in the modern as a result of the contraction between demanding expectations and little energy in parenting. As of March 2020, surveys in 42 countries around the world show that about 5% of parents experience burnout in parenting, with the percentage climbing to 9% in Western countries [3]. The prevalence of parental burnout can even reach higher among parents of children with chronic illnesses [4].

Recent studies have shown that parental burnout can be very destructive. As regards the parents themselves, parental burnout can not only give rise to suicidal and escape ideations [5], but also may lead to external problems such as substance and behavioral addictions and sleep disorders [6]. Prolonged exposure to this negative state results in a significant decrease in the individual's life satisfaction and subjective well-being, and is highly likely to lead to depressive symptoms [7–10]. At the biological level, parental burnout leads to a dysregulation in the hypothalamic-pituitary–adrenal (HPA) axis [11], which is most likely causally implicated in the somatic complaints and sleep difficulties experienced by burnt-out parents and may also be possibly associated with the rise in child-directed aggression [12]. Indeed, in addition to affecting the parents themselves, parental burnout has serious repercussions on children by leading parents to be neglectful or even violent towards their offspring [13, 14]. Parental burnout is also considered to be a risk factor for academic burnout and internal/external problems in children [15–17], which increases adolescents' levels of anxiety and loneliness, aggressive behavior, and depression, reducing adolescents' life satisfaction and mental health [18]. In the case of families, parenting burnout increases the frequency and intensity of spousal conflict [6], strains family relations and reduces the quality of life and life satisfaction of family members.

The concept of burnout was first introduced by Freudenberger in 1974 [19], with the most widely accepted concept proposed by Maslach et al. [20]. One of the conditions that arise when the concept of burnout is applied to the field of parenting is called parenting burnout. According to the Balance between Risks and Resources theory [21], parenting burnout results from an imbalance between excessive parenting demands and limited parenting resources. So far, two instruments to measure parental burnout have been validated. Based on the Maslach Burnout Inventory (MBI) [22], the Parental Burnout Inventory (PBI) firstly developed suggested that parental burnout encompassed three main symptoms: exhaustion related to one's parental role, emotional distancing from one's children, and loss of parental efficiency [23]. Then, the Parental Burnout Assessment (PBA) was developed by Roskam et al. because the PBI may not accurately reflect the experience of burned-out parents [24]. The PBA identified four factors: exhaustion related to one's parental role, emotional distance from one's children, feelings of being fed up with one's parental role, and contrast with how the parent used to and wanted to be [24]. It can be seen that parenting burnout is a unique combination of symptoms, different from parenting stress, burnout and depressive symptoms [9].

The Ecological Systems Theory (EST), developed by American psychologist Urie Bronfenbrenner in 1979, helps to understand the multilevel factors of parenting burnout [25]. The theory holds that subjects and their environment interact in a progressive and reciprocal way to promote individual development. According to the ecosystem theory, the environment that influences health behaviors can be classified into microsystem (individual factors), mesosystem (interpersonal factors), exosystem (organizational or community factors), and macrosystem (society/policy or culture factors). Considering the huge impact of parenting burnout on the three sides of the parent, child, and family as mentioned above, this study aims to systematically assess the associated risk and protective factors for parenting burnout among parents with children aged 0-18 years based on the ecosystem theory. The findings of this study are expected to assist healthcare professionals and policymakers in identifying the mental health requirements of parents who are experiencing parenting burnout and offering them comprehensive care and support.

## Methods

This systematic review was conducted and reported in accordance with the Preferred Systematic Reviews and Meta-Analyses (PRISMA) Statement [26].

### Inclusion and exclusion criteria

To find studies demonstrating relationships between various variables and PB among parents of children including ill aged 0 to 18 in the general population, inclusion and exclusion criteria were established.

The following inclusion criteria for this review were used: (1) any type of observational studies in Chinese or English, including crosssectional studies, cohort studies and case-control studies. (2) conducted between 2010 and 2023. (3) the study reported the association between at least one possible risk or protection factor and PB; PB was reported as the outcome or mediator. (4) a general population sample of parents with children ages 0 to 18 was used for the study.

Exclusion criteria were: (1) study involving new coronavirus background. (2) study is repeated or not available. (3) no extractable factors affecting parenting burnout. (4) grey literature such as expert opinions, conference presentations, dissertations, research and committee reports, and ongoing research.

### Search methods

A systematic search was performed on the electronic databases such as PubMed, Web of Science, EBSCO, CNKI and WanFang database from 2010 to July 2023 for peer-reviewed articles that met the inclusion criteria. We use the search strategy with combinations of the following keywords such as “parenting”, “parental”, “burnout”, “psychological burnout”, “burn-out syndrome”. The search items were connected in PubMed, Web of Science, EBSCO by boolean logic word “AND” and “OR”. The mentioned keywords were also searched in the Chinese language in China electronic databases (CNKI,WanFang database). Each database's search approach was customized, as shown in Table S1. We exported all identified studies and managed by a citation management program (EndNote version X9). Title and abstract screening were performed by two reviewers independently to determine the eligibility of each study. Two reviewers retrieved pertinent publications for full-text reading and subsequent analysis. Consensus was eventually obtained when disagreements were explored with a third reviewer.

### Data extraction and synthesis

One reviewer extracted and arranged the data from each study using an extraction form, and another reviewer confirmed it. First author, publication year, study nation, study design, population and characteristics, sample size and demographic data, PB instruments used, the studied factors, the reported associations between the studied factors and PB, children of the studied parents, PB score were all included in the extracted information. The stated association between the variables and PB at the same time point was collected from cross-sectional studies. The evidence for a relationship between certain parameters and PB was compiled using non-quantitative data synthesis.

### Risk of bias assessment

The included study' quality was evaluated critically using the Quality Assessment Tool for Studies with Diverse Designs (QATSDD), which enables researchers to compare studies with various research designs [27]. Each of the 16 items used by the QATSDD test is evaluated using a 4-point Likert scale that spans from 0 to 3 (0 = not at all, 1 = very slightly, 2 = moderately, 3 = complete; n/a = not applicable). To evaluate the caliber of the included research, the acquired scores were added up and expressed as a percentage of the highest score attainable. Articles with scores over 80% were considered to be of good quality, those with scores between 50 and 80% were considered to be of medium quality, and those with scores below 50% were considered to be of low quality. Evaluation of study quality were performed by two researchers independently and disscussed with inconsistent results.

## Results

### Study selection

**Fig. 1:**
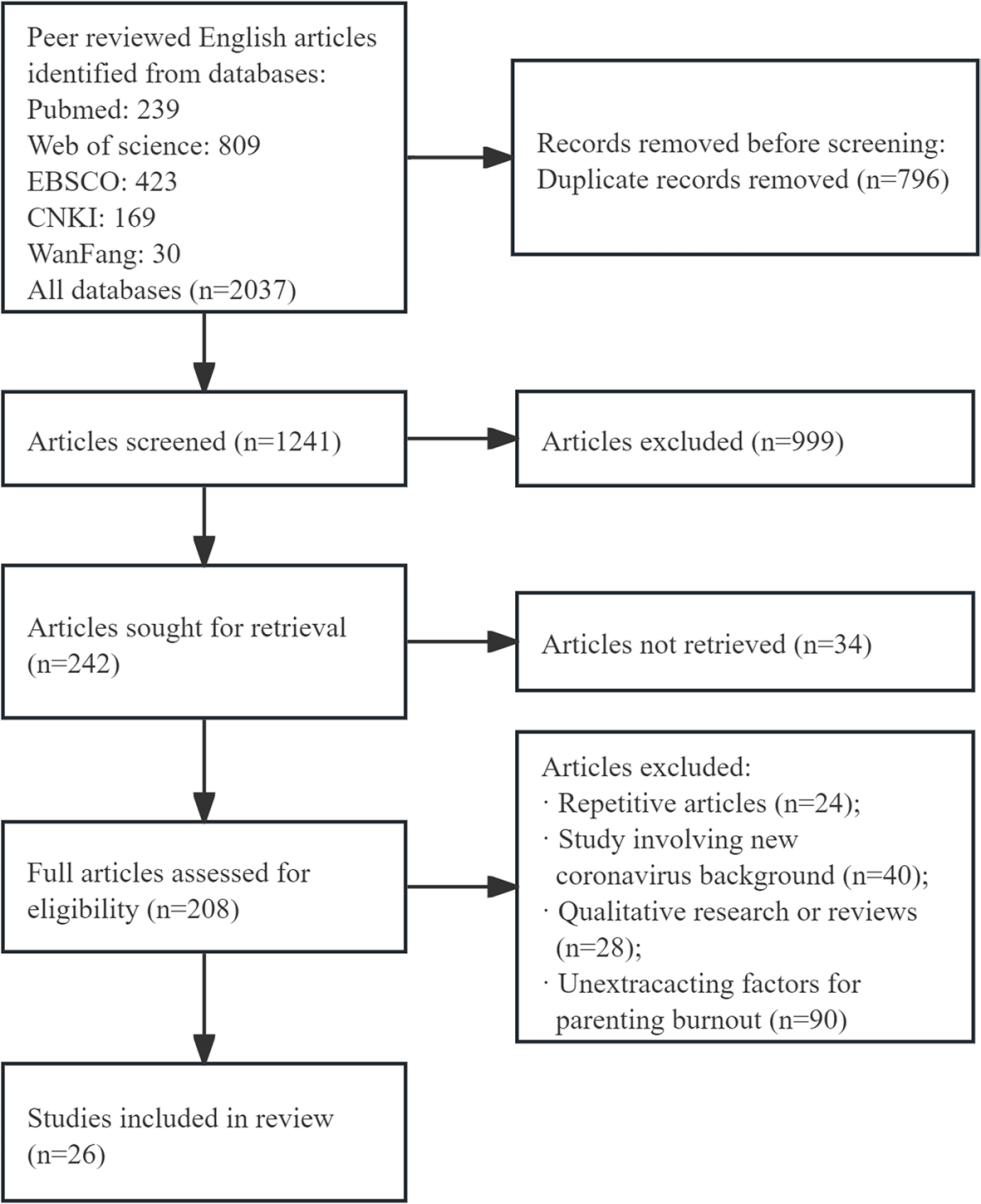
Flow diagram of the study selection process

2037 peer-reviewed publications that were found in the original search were imported into Endnote. 1241 articles were discovered to be potentially pertinent to the research topic after duplicates were eliminated. 242 articles were obtained after the potentially pertinent articles were screened. The total was whittled down to 208 based on the screening of abstracts and titles. 26 studies were included in the review after full texts were examined for eligibility. Figure 1 shows the PRISMA flowchart for the literature search.

### Study characteristic

Table 1 presents the characteristics of the included articles. A total of 1229,128 parents were systematically reviewed in 26 cross-sectional studies [3, 28–52], most of whom were mothers. The studies originated from different countries: China (*n* = 8), Poland (*n* = 4), French (*n* = 2), Switzerland (*n* = 1), Israel (*n* = 1), Africa (*n* = 1), Japan (*n* = 1), Austrian (*n* = 1), Vietnameses (*n* = 1), Lebanese (*n* = 1), Sweden (*n* = 1), Turkey (*n* = 1). There were also combined studies between countries: United Kingdom or United States (*n* = 1), United States, Poland, Peru, Turkey and Belgium (*n* = 1), 42countries (*n* = 1). Twenty-five studies were published between 2018 and 2023, and only one study [48] was published at 2011. The sample sizes ranged from 91 to 1,7409. Ages of the parents ranged from 19 to 65. Children ranged in age from 0 to 18, with the majority being typical between 0 and 6 years old. Two researchs study on parenting burnout in infants [30, 34], five studies [16, 18, 29, 33, 51] on parenting burnout in parents of exceptional children, one study [48] on children with diabetes and one study on kids who needed ongoing pediatric outpatient care [32]. Seventeen research included both mothers and fathers, and nine studies [28, 30, 31, 34, 40–42, 47, 52] were conducted on a sample of just mothers from the general community. The most frequently used measurement for PB was the Parental Burnout Assessment (PBA) with different versions, followed by the Parental Burnout Inventory (PBI) [32, 37, 47, 50], Parental Burnout Measure (PBM-12) [40], Burn-out Measure Short version (BMS-10) [41], Shirom-Melamed Burnout Questionnaire (SMBQ) [48], Maslach Burnout Inventory (MBI) [51].

**Table 1:** Characteristics of the reviewed studies

First author (year)	Location	Sample Size and Age (Mean ± SD)	Questionnaire	Key results	The studied parents’children and children’s age (Mean ± SD)	Score M(SD) (X ± S) or M (P25, P75)
Zhou et al (2023) [28]	China	*N* = 413 All female 22-40 years: 371 (89.8%); 41-57 years: 42 (10.2%)	Self-Regulation Questionnaire-Academic; Parental Burnout Assessment (PBA); Material and Time Richluence Scale (MATAS)	Subjective time pressure plays a mediating role between the internalization of maternal parental motivation and parental burnout, while the employment status of mothers plays a moderating role	At least a child was enrolled in the kindergarten or primary school years	M (SD): 1.96 (0.81)
Zhang et al. (2023) [29]	China	*N* = 723 Female: 489 (67.6%), male: 234 (32.4%) Female: (29.11 ± 1.64); Male: (33.33 ± 1.55)	Parental Burnout Assessment (The Chinese version)	The mediating effect of parenting expectation on personality (neuroticism, agreeableness, and conscientiousness) and parenting burnout of exceptional children was moderated by the gender of parents	Exceptional children Girl: 205; boy: 518, average age (7.24 ± 1.33)	\
Li et al. (2023) [30]	China	*N* = 215 All female	Unmitigated Communion Scale (UCSP); Self-Compassion Scale Short Form (SCS-SF); Parental Burnout Assessment (PBA)	The unmitigated communion not only directly predicts their parental burnout but also indirectly influences parental burnout through self-compassion among infants' mothers	Infants aged 0–3 years old; boy: 96 (44.7%), girl:119 (55.3%). A total of 20 (9.3%) were infants aged 0-1 years, 76 (35.3%) aged 1-2 years, and 119 (55.3%) aged 2–3 years	M (SD): 2.02 (0.64)
Zhuo et al. (2023) [31]	China	*N* = 107 All female (32.83 ± 3.75)	Child-Parent Relationship Questionnaire; Parenting Burnout Scale; Beck Depression Instrument (BDI)	Association between Parent-Child Relationship and Second-Time Mother's Prenatal Depressive Symptoms: The Mediation Role of Parenting Burnout	The firstborns’ average age was 84 months (7.24 ± 2.67)	M (SD): 2.15 (1.17)
Zach et al. (2021) [32]	Israel	*N* = 91 Female:70 (76%), male:21 (24%) (40.3 ± 6.2)	Parental Burnout Inventory; Basic Psychological Needs Scale (BPNS)	Self-compassion and concern for others each predicted PB levels	Children with medical conditions that require chronic pediatric ambulatory treatment (9.6 ± 5.4) years	\
Lin et al. (2023) [33]	China	*N* = 203 Female:136 (67%), male:67 (33%) < 30 years: 50 (24.6%) 30-40 years:127 (62.6%) > 40 years: 26 (12.8%)	Chinese version of the Parenting Burnout Assessment scale; Multidimensional Scale of Perceived Social Support (MSPSS); 20-Item Toronto Alexithymia Scale (TAS-20)	There is a negative association between alexithymia with parental burnout, while perceive social support was the negative predictor of alexithymia and parental burnout. Women have a greater sense of parental burnout than men; The higher the monthly income, the lighter the sense of parental burnout	Autistic children ≤ 3 years:30 (14.8%),3- 6 years:140 (69%),3- 6 years:33 (16.3%)	61 (40, 92.5)
Huang et al. (2023) [34]	China	*N* = 560 All female (30.8 ± 4.8)	Parental burnout assessment (PBA); Edinburgh postnatal depression scale (EPDS)	Postnatal depressive symptoms were positively associated with parental burnout	Infants (9.0 ± 3.2) months	\
Lin et al. ( 2022) [35]	Polish	*N* = 643 Female: 387 (60.2%), male:256 (39.8%) (37.65 ± 7.08)	Polish version of the Twenty Item Values Inventory (TwIVI) questionnaire; Parental Burnout Assessment	Consistent with previous studies, fathers reported fewer parental burnout symptoms compared to mothers.The role of values in predicting parental burnout	At least one child (6.63 ± 5.27) year	M (SD): 31.34 (26.47)
Sodi et al. (2020) [36]	African	*N* = 738 Female:360 (48.8%), male: 378 (51.2%) (38.1 ± 9.63)	Parental burnout assessment (PBA)	Significant association between parental burnout and education level, the number of children in the household, neighborhood, and the number of hours spent with children	Children (5.40 ± 5.92)year	\
Kawamoto et al. (2018) [37]	Japan	*N* = 1200 Female: 600 (50%), male: 600 (50%) (44.1 ± 7.5)	PBI; the Japanese Burnout Inventory; the Japanese version of the Multidimensional Perfectionism Scale; the Japanese version of the Multidimensional Perfectionism Scale; the Todai Health Index Depression Scale	The present study confirmed preliminary validity of the PBI-J and found that parental perfectionism is one of the vulnerability factors in parental burnout	At least one child	The prevalence of parental burnout was estimated to be 4.2 (i.e., PBI-J scores above 88) to 17.3% (i.e., PBI-J scores above 67) in Japan
Szczygieł et al. (2020) [38]	Poland	*N* = 2130 Female:1328 (62.3%), male: 802 (37.7%)	PBA; Personality Inventory NEO-FFI; the Trait Emotional Intelligence Questionnaire-Short Form; the twelve-item version of the Interpersonal Support Evaluation List et al	Significant correlations were found between PBA-PL and neuroticism, emotional intelligence, maladaptive perfectionism, perceived social support, depressive symptoms, marital satisfaction, and life satisfaction	At least one child under the age of 5	Prevalence of parental burnout of 6.8% (9.3% of mothers, 2.3% of fathers) or 4.5% (6.3% of mothers, 1.4% of fathers) on the cut-off score of 86 and 92
Liu et al. (2023) [39]	China	*N* = 249 Female:118 (47.4%), male: 131 (52.6%) (33.95 ± 7.6)	The Parenting Stress Index-Short Form, Parental Burnout Assessment, and Connor-Davidson Resilience Scale	Both the effect of parenting stress on parental burnout and the mediating effect of resilience are moderated by rural/urban residence. This study highlights parenting stress is a risk factor for parental burnout and resilience is the potential mechanism underlying this relation	ASD < 18	M (SD): 22.912 (8.008)
Sekułowicz et al.(2022) [40]	Poland	*N* = 410 All female (39.03 ± 7.42)	Parental Burnout Measure (PBM-12), International Personality Item Pool-Big Five Markers (IPIP-BFM-20), Flexibility and Cohesion Evaluation Scales (FACES-IV), a survey on childcare difficulties	These findings suggest that increased maternal emotional instability (neuroticism) and conscientiousness can lead to increased family communication problems, which may further lead to a breakdown of the equilibrium in the family system, resulting in the mother’s dissatisfaction with family life and a consequent increased risk of maternal burnout	ASD (9.74 ± 7.41)	M (SD) 28.03 (6.62)
Séjourné et al. (2018) [41]	French	*N* = 263 All female 20-49 years	Burn-out Measure Short version (BMS-10), the Multidimensional Scale of Perceived Social Support (MSPSS), the Parenting Stress Index Short Form (PSI-s), the Hospital Anxiety and Depression Scale (HADS), the Bromley postnatal depres sion scale	A clear relationship between maternal burn-out and anxiety and depressive symptoms as well as parental stress	Children’s ages ranged from 0-17 years: (4 ± 3.85)	20% of mothers were affected by maternal burn-out
Roskam et al. (2021) [3]	42 countries	*N* = 17,409 Female: 12,361 (71%), male: 5048 (29%) Mean age: 39.20	Parental burnout assessment (PBA)	Cultural values in Western countries may put parents under heightened levels of stress	At least one child	\
Prandstetter et al. (2023) [42]	Austrian	*N* = 121 All female (38.9 ± 7.5) min = 25, max = 55	Parenting Scale (PS); Couple Satisfaction Index (CSI-4); Intimate Partner Violence victimization (IPV victimization); Parental Burnout Assessment (PBA) et al	Mothers' self-reports on PB were significantly linked to couple dissatisfaction and negative parenting behaviors. Decreasing couple conflict and violence may help to reduce parental stress, which in turn could minimize the risk of developing PB	Children aged 0-17 years	\
Piotrowski et al. (2023) [43]	Polish	*N* = 1471 Female:1199 (81.5%), male: 265 (18%) 7 individuals who identified their gender as nonbinary or did not want to provide gender information (0.5%) 19 to 45 years ( 35.30 ± 5.98)	23-item Parental Burnout Assessment; a shortened version of the Formal Characteristics of Behaviour-Temperament Inventory; the International Personality Item Pool-Big Five Markers-20 (IPIP-BFM-20); the Utrecht-Management of Identity Commitments Scale (U-MICS)	The severity of parental burnout was linked to traits ranging from biologically determined temperament traits to basic personality traits to a sense of parental identity	1 month to 26 years (8.22 ± 6.21)	\
Hong et al. (2022) [44]	Vietnamese	N = 821 Female:657 (80%), male:164 (20%) (39.49 ± 5.56)	Vietnamese version of the Strengths and Difficulties Questionnaire; the Vietnamese version of the Parental Burnout Assessment (PBA); the Self-compassion Scale-Short Form (SCS-SF)	Primary students' behavior problems were significantly positively correlated to parental burnout while negatively correlated with parents' self-compassion and academic outcomes	Students (10.12 ± 0.853)	M (SD): 35.3 (18.354)
Favez N et al. (2023) [45]	Switzerland	*N* = 306 Female: 120 (39.2%), male: 186 (60.8%) (39.71 ± 6.56)	Coparenting Relationship Scale; Parental Burnout Assessment	A higher number of children and having younger children are linked to higher burnout; coparenting exposure to conflict is related to higher burnout, whereas endorsement of the partner's parenting is related to lower burnout	Children (5.26 ± 4.73)	\
Gannagé et al. (2020) [46]	Lebanese	*N* = 200 Female: 134 (67%), male: 66 (33%) 20-55 years (37.51 ± 8.40)	Parental Burnout Assessment	There was no significant gender difference in the prevalence of parental burnout, but mean levels were higher in mothers than in fathers. Both less educated parents and single parents reported higher parental burnout	(10.56 ± 8.02) for the oldest and (6.74 ± 5.86) for the youngest	Depending on the cut-off score used (i.e., 76 or 92), the analyses yielded a prevalence of parental burnout of 6.5% (7.5% among mothers, 4.5% among the fathers) or 5.5% (6.7% among mothers, 3% among the fathers), respectively, in Lebanese parents
Meeussen et al. (2018) [47]	United Kingdom or United States	*N* = 169 All female (36.74 ± 7.62)	Parental Burnout Inventory	Feeling pressure to be a perfect mother was positively related to parental burnout, and this relation was mediated by parental stress, by a stronger cognitive prevention focus aimed at avoiding mistakes as a mother, and by higher maternal gatekeeping behaviors taking over family tasks from one's partner	Child ranging from zero to 20 (6.68 ± 5.49)	M (SD): 3.34 (1.44)
Lindström et al. (2011) [48]	Sweden	*N* = 252 Female: 142 (56.3%), male: 109 (43.7%) Female: 44 (30-56), male: 42 (25-56)	Shirom-Melamed Burnout Questionnaire (SMBQ); Performance-Based Self-Esteem scale (PBSE)	For both genders, parental burnout was associated with low social support, lack of leisure time, financial concerns and a perception that the child’s disease affects everyday life. Low self-esteem and high need for control were risk factors for maternal burnout	The mean age of the children at the onset of T1DM was 7.5 (0.9-15.5) years, and the mean duration of their disease was 5.4 (0.5-16.5) years	\
Lin et al. (2022) [49]	United States, Poland, Peru, Turkey and Belgium	*N* = 1835 Belgium: (40.45 ± 8.98), Peru: (42.11 ± 13.01), Poland: (36.50 ± 7.87), Turkey: (35.63 ± 5.73), USA: (36.98 ± 8.75), Total: (37.38 ± 8.7)	12-item Interpersonal Support Evaluation List; the reappraisal subscale of the Emotion Regulation Questionnaire (ERQ); Parental Burnout Assessment	Both social support and cognitive reappraisal were associated with lower parental burnout	At least one child	M (SD): 26.67 (25.31)
Vigouroux et al. (2018) [50]	France	*N* = 372 Female: 314 (84.4%), male: 58 (15.6%) 23 to 65 years (36.76 ± 7.57)	Parental Burnout Inventory; the Alter Ego; the French translation of the Ten-Item Personality Inventory	The number of children and wide age gaps between these children were moderate risk factors for parental burnout syndrome; emotional stability (opposite of neuroticism) was the personality trait that afforded the greatest protection against parental burnout syndrome; the same three personality traits (neuroticism, agreeableness, and conscientiousness) were linked to parental burnout and its three dimensions	First child between the ages of 19 and 47 (29.66 ± 4.44)	\
Meryem et al. ( 2021) [51]	Turkey	145 children with ASD and 127 control children were enrolled along with their mothers and fathers Mean ages of mothers and fathers of children with ASD were (34.9 ± 6.5) and (38.4 ± 9.0) years, respectively, while the corresponding ages for parents of children in the control group were (37.4 ± 6.2) and (39.2 ± 11.4) years	Beck Depression Inventory (BDI); Maslach Burnout Inventory (MBI); Childhood Autism Rating Scale (CARS)	Both mothers and fathers of children with ASD reported significantly elevated depressive and burnout symptoms; burnout scores of mothers were greater than those of fathers	145 children with ASD (7.2 ± 3.8) and 127 control children (9.5 ± 4.2)	\
Cheng et al. (2021) [52]	China	*N* = 227 All female (40.52 ± 3.93)	Parental Burnout Assessment (PBA); Adult Attachment Scale (AAS); Simplified Coping Style Questionnaire (SCSQ)	Mother's attachment style not only affects their parental burnout directly, but also affecst their parental burnout indirectly through the mediation effect of negatively coping style	Grade 8 students	M (SD): 2.03 (0.94)

### Methodological quality of included study

According to Table S2, the QATSDD evaluation found that twenty-two included studies were of medium quality in this systematic review, three [30, 43, 50, 52] were low quality and one was high quality [43]. Scores from the QATSDD ranged from 40.5% to 83.3%, with a mean score of 62.17%.

### Parental burnout in parents with at least a child

The mean score of parental burnout in parents with at least a child based on PBA using the Likert 5 points [28, 30, 52] was 2.00 (SD = 0.80), based on PBA using the Likert 7 points [35, 39, 44, 49] was 29.05 (SD = 19.53), based on PBS [31]was 2.15 (SD = 1.17), based on PBM-12 [40] was 28.03 (SD = 6.62) and based on PBI [47] was 3.34 (SD = 1.44).

### Factors associated with parental burnout

**Fig. 2:**
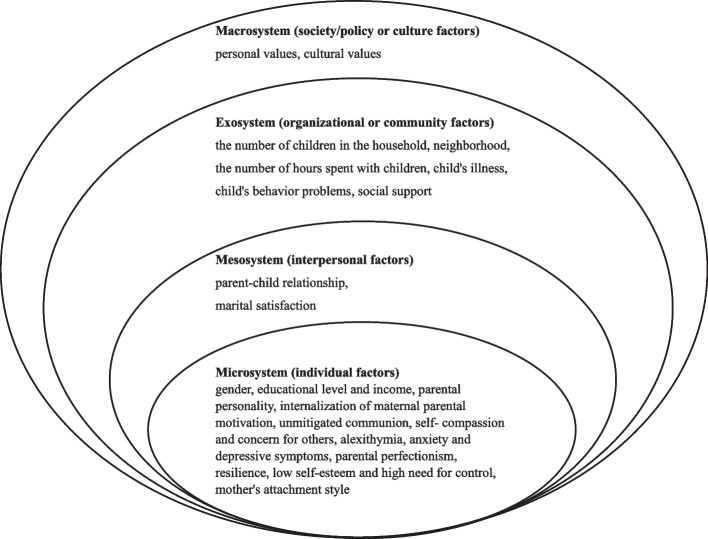
Framework diagram of influencing factores of parental burnout

Based on the social-ecological system theory, this study categorized the factors affecting parenting burnout into microsystem (individual factors), mesosystem (interpersonal factors), exosystem (organizational or community factors), and macrosystem (society/policy or culture factors). This study constructed a framework diagram of influencing factors of parental burnout in Fig. 2.

### Microsystem-individual factors

As far as parents are concerned, parenting burnout is influenced by their own factors to some extent. General demographic factors associated with parental burnout were gender, educational level and income [42]. Mothers are more likely to experience parenting burnout than fathers [35, 46, 51]. Less educated parents and single parents reported higher parental burnout [36, 46]. The higher the monthly income, the lighter the sense of parental burnout [33]. Parental factors such as parental personality (neuroticism, agreeableness and conscientiousness) [29, 40, 43], internalization of maternal parental motivation [28], unmitigated communion [30], self-compassion and concern for others [32], alexithymia [33], anxiety and depressive symptoms [34], parental perfectionism [37, 47], resilience [39], low self-esteem and high need for control [48], mother's attachment style [52] had a significant relationship with parental burnout among parents with at least a child.

### Mesosystem-interpersonal factors

For parent–child relationship, a poor parent–child relationship could result in parenting burnout, while a good parent–child relationship can positively affect parenting burnout [31]. For interpersonal factors in spouses, marital satisfaction in demographic information is related to parenting burnout [42]. Satisfaction with marital status stems from the establishment of good interpersonal relationships between couples, and couples with good marriages share parenting responsibilities, provide timely emotional support to each other, and maintain good communication and interaction styles, resulting in low parenting stress and burnout.

### Exosystem-organizational or community factors

Significant association between parental burnout and the number of children in the household, neighborhood and the number of hours spent with children [36]. A higher number of children and having younger children are linked to higher burnout among parents [45, 50]. Beyond that, childhood illness is also a risk factor for parenting burnout. In the case of children with autism [33, 39], for example, the social and communication barriers associated with the disease lead to parenting burnout by affecting the interpersonal relationships between parents and children. Children’ behavior problems and negative parenting behaviors were significantly positively correlated to parental burnout by affecting parent–child communication [42, 44]. Furthermore, social support as an organizational or community factor strongly protected parental burnout from parents [38, 48, 49].

### Macrosystem-society/policy or culture factors

The role of personal values in predicting parental burnout [35]. When parents prioritized the individualistic ideals of power and achievement, which emphasize personal success by demonstrating competence in accordance with existing cultural standards, they were more likely to have PB symptoms. In contrast, parents who placed a higher priority on benevolence (which emphasizes the maintenance and improvement of the wellbeing of those with whom they frequently interact) of collectivism saw fewer symptoms of PB. In terms of cultural values,cultural values in western countries may put parents under heightened levels of stress [3].

## Discussion

With this systematic review, we wanted to present a summary of the research on the variables related to general parental burnout (PB) among parents of children in the general population aged 0 to 18 years old. There were 26 studies listed in all. Overall, a cross-sectional design was used in the great majority of research including mothers. Parental burnout was found to be favorably or negatively correlated with four categories of factors, such as microsystem-individual factors (gender, educational level, income, parental personality, internalization of maternal parental motivation, unmitigated communion, self-compassion and concern for others, alexithymia, anxiety and depressive symptoms, parental perfectionism, resilience, low self-esteem and high need for control, mother's attachment style), mesosystem-interpersonal factors (parent–child relationship and marital satisfaction), exosystem-organizational or community factors (the number of children in the household, neighborhood and the number of hours spent with children, child's illness, child's behavior problems, social support) and macrosystem-society/policy or culture factors (personal values and cultural values).

Since the family is the site of parenting and parents are important members of the family, factors within the parents themselves may play a role in parenting burnout by increasing/decreasing the resources they need to raise their children. These factors include neuroticism, agreeableness and conscientiousness. Neuroticism is one of the most important risk factors in parental burnout. Researchers have found that neuroticism (emotional instability) affects emotion regulation and impulse control and emotionally unstable parents are more reactive to life events more likely to lead to burnout. This is consistent with the results of the meta-analysis by Alarcon et al. [53]. Agreeability refers to attributes beneficial to the child, which was inversely correlated with parental burnout. High parental agreeableness predicts higher levels of positive and flexible cognitive coping strategies and lower levels of avoidant cognitive strategies, emphasizing the maintenance of positive parent–child interactions and positive feedback from the child's perception of being a "capable" parent, which reduces parenting burnout [29]. Conscientiousness encompasses a tendency for meticulousness and obsessiveness as well as self-control, organization and planning-all of which are presumably beneficial traits. Maternal burnout was negatively correlated with conscientiousness. Individuals with higher levels of conscientiousness reported fewer negative effects and were better able to automatically down-regulate parenting burnout.These results suggest that traits of a resilient personality are associated with lower parental [54] burnout [39, 54].

Unmitigated communion is a personality trait that involves excessive focus on others to the exclusion of the self and is associated with female gender roles. Unmitigated communion is positively associated with negative interpersonal interactions and social vulnerability and oversharing individuals not only face more peer conflict [55, 56], but are also more sensitive to conflict in their relationships. It can lead to persistent negative emotions, which undoubtedly increase parenting burnout [57].

The degree of internalization of maternal parental motivation significantly and negatively predicts parenting burnout. The self-determination theory suggests that autonomous motivation is conducive to positive individual development, while controlled motivation is detrimental to positive individual development [56]. If mothers are motivated to raise their children based on interest, they will feel more positive emotions and experience more meaning in the process of parenting. Therefore, they will experience less parenting burnout. This is consistent with the findings of self-determination theory in the areas of exercise, work [58, 59].

Self-compassion and concern for others can reduce burnout among caregivers. This is similar to the findings of two studies [60, 61]. Prior research has shown that both caring for others and self-compassion are beneficial in promoting well-being [62, 63]. It is worthy that PB reflects a reduction in parental well-being. Research based on self-determination theory shows that a self-compassionate person will nourish his need for autonomy and that need fulfillment is associated with reduced burnout [64]. The basic need for relatedness is nurtured by self-expression in the form of concern for others, which in turn lessens burnout [64].

Alexithymia is a risk factor for parenting burnout in parents of children with autism. People with high levels of alexithymia feel more psychological stress and are more likely to intrinsically use ineffective methods such as avoidance and self-blame to mask the stress [65, 66], so they feel more burnout. According to studies [67], mindful meditation may lessen alexithymia by changing how physical and emotional experiences are perceived. In order to help parents of autistic children, health practitioners are encouraged to offer courses on mindfulness-based stress reduction. Additionally, alexithymia can be efficiently changed by enhancing emotional expression. In order to decrease parental burnout, it is possible to cultivate parents' emotional sensitivity through arts learning [68, 69].

Postpartum depressive symptoms were positively associated with parental burnout, a finding that is consistent with previous studies of mothers raising older children [41]. Parents with depressive symptoms are mentally unstable when dealing with parenting-related issues and may be prone to negative emotions such as self-denial. In addition, they are reluctant to address issues related to their children, are psychologically and behaviorally distant from their children, which are more prone to parenting burnout [34].

Parental perfectionism is a risk factor for parenting burnout. A recent meta-analysis present the strength of the correlations between perfectionism and burnout [70]. Recent research has shown that parenting perfectionism increases the use of expressive repression, which ultimately exacerbates PB [71]. Perfectionism predisposes parents to experience frequent worry and strong negative emotions, to set impossibly high standards for themselves while being overly critical of their own actions and mistakes and to be more likely to experience parenting burnout [36].

Maternal attachment styles were associated with parenting burnout, with anxious attachment styles positively associated with parenting burnout and close-dependent attachment styles negatively associated with parenting burnout. Mothers with high anxious attachment are more likely to experience increased negative emotions and negative interactions with their parents and children when faced with the stress of parenting activities, which can lead to feelings of emotional exhaustion and emotional detachment from their children, leading to parenting burnout [72]. Whereas the close-dependent attachment style can be used as an internal resource to help individuals cope effectively with life's difficulties, individuals with anxious attachment tend to use negative coping styles when facing stressful situations [72].

Our findings identified sociodemographic factors that predicted PB including parental gender, educational level, income, marital satisfaction and life satisfaction. Mothers reported more PB symptoms compared to fathers, consistent with the findings of han et al. [73]. Possible reasons are influenced by the traditional concept of the family, in which mothers bear the main responsibility for the care and education of their children. Higher levels of literacy are associated with lower levels of parenting burnout. Parents with a high level of education can approach the stress of parenting in a more logical way, adopt scientific methods to seek assistance and deal with issues that arise during the parenting [73]. Economic status is an important factor in parenting burnout, especially for parents of sick children, and an adequate monthly income can pay for treatment and reduce the burden of parenting [50]. Therefore, it is recommended that the government should increase their welfare benefits and reimbursement of treatment costs. Based on Bronfenbrenner's social-ecological systems theory of mesosystems, family conflict and marital satisfaction symbolize the interpersonal relationship between family members. Apparently, the family is the environment to which children have the most contact. Family dysfunction, such as higher levels of family disintegration and conflict and lower levels of marital satisfaction, are associated with a higher risk of parental burnout. Therefore, in order to reduce parental burnout, more attention should be paid to parental burnout among mothers with low levels of education and marital/life satisfaction in the family.

It is worth noting that parenting burnout is evident among parents of sick children and children who require ongoing pediatric outpatient care, especially parents of exceptional children. In the case of parents of children with autism spectrum disorders (ASD), the need for long-term care for the child, changes in family roles and daily routines, difficulties encountered during diagnosis and access to services, lack of diagnostic information, the burden and fatigue associated with the urgent need for pertinent information about educational and rehabilitative services, the financial burdens imposed by the child's educational and rehabilitative services and the difficulty for parents to participate in the social life, are among the range of issues that can cause parents of children with ASD experience tremendous parenting stress [74]. According to the Risk-Resource Balance Theory [21], parenting burnout occurs when stressors accumulate to a certain level without adequate resources and external support to compensate and intervene, i.e., when parents chronically lack the parenting resources needed to cope with specific parenting stresses. So for children with diseases, especially parents of children with ASD, parents should be the focus of medical institutional. Social support reduces parenting burnout, meaning that parents are less likely to burn out if they have people around them with whom they can engage in a variety of activities and from whom they can get advice or material help when needed. Therefore, we call on spouses to solve problems by helping each other, affirming each other's parenting skills, respecting each other's contributions, supporting each other's authority and parenting decisions and other ways to give social support to alleviate parents' parenting burnout [75]. In addition, the construction of a multi-dimensional support system with the participation of multiple parties, including family, school, community and government, is also recommended.

Our findings demonstrated that personal values contribute to PB. Parents from individualistic countries seem to be particularly vulnerable. According to Roskam 's study, it also show the association between the mean levels of PB and group tendency of individualism-collectivism cultural value across countries [76]. Western parents are five times more likely to experience this syndrome than non-Western parents. Even after accounting for economic disparities between nations, as well as individual and family variables, western parents still have a stronger group tendency of individualism (lower tendency of collectivism) cultural value. Mechanisms linking individualism and parental burnout remain to be investigated. At present, affective mechanisms or parental emotion regulation seems to be a prominent candidate. Strengthening social networks of family mutual aid and solidarity may help reduce the prevalence of parental burnout in individualistic countries.

Some limitations remain in our systematic review. First, due to the inclusion and exclusion criteria utilized, some studies may have been missed despite the well coordinated effort to thoroughly search the literature. Second, only peer-reviewed English-language and Chinese-language studies that had been published during the previous 13 years were included in the review. Third, a meta-analysis was not possible due to the methodological and instrumental variability in this review. Lack of meta-analysis can result in inconsistent results, but the present study's rigorous approach to data collecting, sorting and analysis of trials held up well. Fourth, causalities cannot be ascertained as all of the studies followed a cross-sectional design.

### Implications for future research

Based on the results of the current systematic review, it is recommended that future research pay more attention to the factors associated with parental burnout in parents with children. Longitudinal studies are recommended to evaluate the associations of factors with PB. Future research should specifically address the exploration of factors influencing parenting burnout in children with specific disorders, such as ASD. It is suggested that researchers may be guided by a theoretical framework when considering parenting burnout factors when developing research designs. Most of the studies included in this review focus on mothers, who are influenced by the traditional thinking that mothers take the main care of their children. In the last few decades, fathers have taken a more active role in caring for their children [77]. In the future, it is recommended that researchers focus on the current state of parenting burnout and the factors influencing it in this group of fathers.

## Conclusions

Parenting burnout is an important issue that affects children, families and society, as well as parents' own quality of life. This study summarizes the evidence that individual factors, interpersonal factors, organizational or community factors and society/policy or culture factors of children's parents are associated with PB. The microsystem-individual factors such as gender, educational level, income, parental personality, internalization of maternal parental motivation, unmitigated communion, self-compassion and concern for others, alexithymia, anxiety and depressive symptoms, parental perfectionism, resilience, low self-esteem and high need for control, mother's attachment style. The mesosystem-interpersonal factors are mainly parent-child relationship and marital satisfaction. The exosystem-organizational or community factors include the number of children in the household, neighborhood, the number of hours spent with children, child's illness, child's behavior problem and social support. Macrosystem-society/policy or culture factors are mainly personal values and cultural values. These modifiable variables are available to support child health care and social professionals. It is suggested that future longitudinal studies could look more closely at factors associated with PB based on socio-ecological theory to inform the development of intervention strategies.

### Supplementary Information

**Additional file 1:  Table S1.** Searching strategies. **Table S2.** Quality appraisal of the reviewed studies.

## Data Availability

All data generated or analysed during this study are included in this published article [and its Supplementary information files].

## Abbreviations

PB: Parental burnout
EST: Ecological systems theory
MBI: Maslach burnout inventory
PBI: Parental burnout inventory
PBA: Parental burnout assessment
PRISMA: Preferred systematic reviews and meta-analyses
QATSDD: Quality assessment tool for studies with diverse designs
ASD: Autism spectrum disorders

## References

[1] Abidin RR (1990). Introduction to the special issue - the stresses of parenting. J Clin Child Psychol.

[2] Mikolajczak M, Raes ME, Avalosse H, Roskam I (2018). Exhausted Parents: Sociodemographic, Child-Related, Parent-Related, Parenting and Family-Functioning Correlates of Parental Burnout. J Child Fam Stud.

[3] Roskam I, Aguiar J, Akgun E, Arikan G, Artavia M, Avalosseet H (2021). Parental Burnout Around the Globe: a 42-Country Study. Affect Sci.

[4] Lindstrom C, Aman J, Norberg AL (2010). Increased prevalence of burnout symptoms in parents of chronically ill children. Acta Paediatr.

[5] Mikolajczak M, Grosse JJ, Roskam I (2019). Parental Burnout: What Is It, and Why Does It Matter?. Clin Psychol Sci.

[6] Mikolajczak M, Brianda ME, Avalosse H, Roskam I (2018). Consequences of parental burnout: Its specific effect on child neglect and violence. Child Abuse Negl.

[7] Van Bakel HJA, Van Engen ML, Peters P (2018). Validity of the Parental Burnout Inventory Among Dutch Employees. Front Psychol.

[8] Zou R, Hong X, Wei G, Xu X, Yuan J (2022). Differential Effects of Optimism and Pessimism on Adolescents' Subjective Well-Being: Mediating Roles of Reappraisal and Acceptance. Int J Environ Res Public Health.

[9] Mikolajczak M, Gross JJ, Stinglhamber F, Norberg AL, Roskam I (2020). Is Parental Burnout Distinct From Job Burnout and Depressive Symptoms?. Cin Psychol Sci.

[10] Sabzi N, Khosravi Z, Kalantar-Hormozi B (2023). Parental burnout and depression among Iranian mothers: The mediating role of Maladaptive Coping modes. Brain Behav.

[11] Brianda ME, Roskam I, Mikolajczak M (2020). Hair cortisol concentration as a biomarker of parental burnout. Psychoneuroendocrinology.

[12] Sarrionandia-Pena A (2019). Effect size of parental burnout on somatic symptoms and sleep disorders. Psychotherapy Psychosomatics.

[13] Chen Y, Haines J, Charlton BM, VanderWeele TJ (2019). Positive parenting improves multiple aspects of health and well-being in young adulthood. Nat Hum Behav.

[14] Mikolajczak M, Roskam I (2021). Conséquences du burn-out parental sur le parent et les enfants [Consequences of parental burnout on the parent and the children]. Soins Pediatr Pueric.

[15] Li Z, Luo J, Song F, Li J, Shen Y. The Relationship Between Parental Burnout and Children's Learning Burnout: A Moderated Chain Mediation Model. Psychol Rep. Published online February 8, 2023. 10.1177/00332941231156810 36754547

[16] Zhang H, Li S, Wang R, Hu Q (2023). Parental burnout and adolescents' academic burnout: Roles of parental harsh discipline, psychological distress, and gender. Front Psychol.

[17] Chen BB, Qu Y, Yang B, Chen X (2022). Chinese mothers' parental burnout and adolescents' internalizing and externalizing problems: The mediating role of maternal hostility. Dev Psychol.

[18] Yuan Y, Wang W, Song T, Li Y (2022). The Mechanisms of Parental Burnout Affecting Adolescents' Problem Behavior. Int J Environ Res Public Health.

[19] Peirson J (2022). Staff burn-out has implications for organisational and patient outcomes: would an open culture of support with structures in place prevent burn-out?. Evid Based Nurs.

[20] Maslach C, Schaufeli WB, Leiter MP (2001). Job burnout. Annu Rev Psychol.

[21] Mikolajczak M, Roskam I (2018). A Theoretical and Clinical Framework for Parental Burnout: The Balance Between Risks and Resources (BR2). Front Psychol.

[22] Soares JP, Lopes RH, Mendonça PBS, Silva CRDV, Rodrigues CCFM, Castro JL (2023). Use of the Maslach Burnout Inventory Among Public Health Care Professionals: Scoping Review. JMIR Ment Health.

[23] Roskam I, Raes ME, Mikolajczak M. Exhausted Parents: Development and Preliminary Validation of the Parental Burnout Inventory [published correction appears in Front Psychol. 2018 Jan 30;9:73]. Front Psychol. 2017;8:163. 10.3389/fpsyg.2017.00163 PMC5298986 28232811

[24] Roskam I, Brianda ME, Mikolajczak M (2018). A Step Forward in the Conceptualization and Measurement of Parental Burnout: The Parental Burnout Assessment (PBA). Front Psychol.

[25] Hua W, Fang Q, Lin W, Liu Z, Lu W, Zhu D (2022). The level and influencing factors of graduating nursing students' professional commitment from the perspective of Ecological Systems Theory: A cross-sectional study. Nurse Educ Today.

[26] Page MJ, McKenzie JE, Bossuyt PM, Boutron I, Hoffmann TC, Mulrow CD (2021). The PRISMA 2020 statement: an updated guideline for reporting systematic reviews. BMJ.

[27] Sirriyeh R, Lawton R, Gardner P, Armitage G (2012). Reviewing studies with diverse designs: the development and evaluation of a new tool. J Eval Clin Pract.

[28] Zhou J, Zhong S, Xu H (2023). Relationship between degree of maternal parenting motivation internalization and parenting burnout: mediation of subjective time stress and modulation of maternal employment status. Chin J Clin Psychol.

[29] Zhang J, Chen Y, Liu Y (2023). Effect of parental personality in special children on parenting burnout: a moderated mediation model. J Huzhou Nor Univ.

[30] Li X, Wang L, Huang J (2023). Excessive sharing and parenting burnout in infants and young mothers: the mediating role of self-care. Chin J Clin Psychol.

[31] Zhuo R, Shi X, Wu Y (2022). Association between Parent-Child Relationship and Second-Time Mother's Prenatal Depressive Symptoms: The Mediation Role of Parenting Burnout. Int J Environ Res Public Health.

[32] Gerber Z, Davidovics Z, Anaki D (2021). The Relationship Between Self-Compassion, Concern for Others, and Parental Burnout in Child's Chronic Care Management. Mindfulness (N Y).

[33] Lin Y, Wang Y, Lin C (2023). The mediating role of perceived social support: alexithymia and parental burnout in parents of children with autism spectrum disorder. Front Psychol.

[34] Huang Y, Mao F, Zhang X, Wang J, Xu Z, Cao F (2023). Exploring the relationship between postnatal depressive symptoms and parental burnout from the perspective of the population and individual level. BMC Psychiatry.

[35] Lin GX, Szczygieł D (2022). Basic Personal Values and Parental Burnout: A Brief Report. Affect Sci.

[36] Sodi T, Kpassagou LB, Hatta O, Ndayizigiye A, Ndayipfukamiye JM, Tenkué JN (2020). Parenting and parental burnout in Africa. New Dir Child Adolesc Dev.

[37] Kawamoto T, Furutani K, Alimardani M (2018). Preliminary Validation of Japanese Version of the Parental Burnout Inventory and Its Relationship With Perfectionism. Front Psychol.

[38] Szczygieł D, Sekulowicz M, Kwiatkowski P, Roskam I, Mikolajczak M (2020). Validation of the Polish version of the Parental Burnout Assessment (PBA). New Dir Child Adolesc Dev.

[39] Liu S, Zhang L, Yi J, Liu S, Li D, Wu D, et al. The Relationship Between Parenting Stress and Parental Burnout Among Chinese Parents of Children with ASD: A Moderated Mediation Model. J Autism Dev Disord. 10.1007/s10803-022-05854-y 36637590

[40] Sekułowicz M, Kwiatkowski P, Manor-Binyamini I, Boroń-Krupińska K, Cieślik B (2022). The Effect of Personality, Disability, and Family Functioning on Burnout among Mothers of Children with Autism: A Path Analysis. Int J Environ Res Public Health.

[41] Séjourné N, Sanchez-Rodriguez R, Leboullenger A, Callahan S (2018). Maternal burn-out: an exploratory study. J Reprod Infant Psychol.

[42] Prandstetter K, Murphy H, Foran HM (2023). The Role of Intimate Partner Violence, Couple Dissatisfaction and Parenting Behaviors in Understanding Parental Burnout. J Child Fam Stud.

[43] Piotrowski K, Bojanowska A, Szczygieł D, Mikolajczak M, Roskam I (2023). Parental burnout at different stages of parenthood: Links with temperament, Big Five traits, and parental identity. Front Psychol.

[44] Hong VTP, An NH, Thao TTP, Thao LN, Thanh NM (2022). Behavior problems reduce academic outcomes among primary students: A moderated mediation of parental burnout and parents' self-compassion. New Dir Child Adolesc Dev.

[45] Favez N, Max A, Bader M, Tissot H (2023). When not teaming up puts parents at risk: Coparenting and parental burnout in dual-parent heterosexual families in Switzerland. Fam Process.

[46] Gannagé M, Besson E, Harfouche J, Roskam I, Mikolajczak M (2020). Parental burnout in Lebanon: Validation psychometric properties of the Lebanese Arabic version of the Parental Burnout Assessment. New Dir Child Adolesc Dev.

[47] Meeussen L, Van Laar C (2018). Feeling Pressure to Be a Perfect Mother Relates to Parental Burnout and Career Ambitions. Front Psychol.

[48] Lindström C, Aman J, Norberg AL (2011). Parental burnout in relation to sociodemographic, psychosocial and personality factors as well as disease duration and glycaemic control in children with Type 1 diabetes mellitus. Acta Paediatr.

[49] Lin GX, Goldenberg A, Arikan G, Brytek-Matera A, Czepczor-Bernat K, Manrique-Millones D (2022). Reappraisal, social support, and parental burnout. Br J Clin Psychol.

[50] Vigouroux SL, Scola C (2018). Differences in Parental Burnout: Influence of Demographic Factors and Personality of Parents and Children. Front Psychol.

[51] Kütük MÖ, Tufan AE, Kılıçaslan F, Güler G, Çelik F, Altıntaş E, et al. High Depression Symptoms and Burnout Levels Among Parents of Children with Autism Spectrum Disorders: A Multi-Center, Cross-Sectional, Case-Control Study [published correction appears in J Autism Dev Disord. 2021 Feb 16;:]. J Autism Dev Disord. 2021;51(11):4086-4099. 10.1007/s10803-021-04874-4 33459915

[52] Cheng L, Wang W, Wang S (2021). Attachment styles and maternal parenting burnout: the mediating role of coping styles. Chin J Clin Psychol.

[53] Alarcon G, KJ Eschleman, NA Bowling. Relationships between personality variables and burnout: A meta-analysis. Work Stress. 2009; 23(3): 244-263.

[54] Caspi A, Roberts BW, Shiner RL (2005). Personality development: stability and change. Annu Rev Psychol.

[55] Bruch MA (2002). The relevance of mitigated and unmitigated agency and communion for depression vulnerabilities and dysphoria. J Couns Psychol.

[56] Helgeson VS, Palladino DK (2012). Agentic and communal traits and health: adolescents with and without diabetes. Pers Soc Psychol Bull.

[57] Reynolds KA, Helgeson VS, Seltman H, Janicki D, Page-Gould E, Wardle M (2006). Impact of interpersonal conflict on individuals high in unmitigated communion. J Appl Soc Psychol.

[58] Deci EL, Ryan RM (2000). The "what" and "why" of goal pursuits: Human needs and the self-determination of behavior. Psychol Inq.

[59] Chen W, Gan Y, Guo Z (2014). The relationship between meaning thinking and work burnout from the perspective of self-determination theory. Chin J Clin Psychol.

[60] Pereira AT, Brito MJ, Cabaços C, Carneiro M, Carvalho F, Manão A (2022). The Protective Role of Self-Compassion in the Relationship between Perfectionism and Burnout in Portuguese Medicine and Dentistry Students. Int J Environ Res Public Health.

[61] Gerber Z, Tolmacz R, Doron Y (2015). Self-compassion and forms of concern for others. Personality Individ Differ.

[62] Gunnell KE, Mosewich AD, McEwen CE, Eklund RC, Crocker PRE (2017). Don't be so hard on yourself! Changes in self-compassion during the first year of university are associated with changes in well-being. Personality Individ Differ.

[63] Zessin U, Dickhäuser O, Garbade S (2015). The Relationship Between Self-Compassion and Well-Being: A Meta-Analysis. Appl Psychol Health Well Being.

[64] Hashem Z, Zeinoun P (2020). Self-Compassion Explains Less Burnout Among Healthcare Professionals. Mindfulness (N Y).

[65] Wu C, Shi C, Dong W, Li B, Wu R (2020). Association Between Alexithymia and Immature Coping Styles Is Mediated by Self-Inconsistency and Is Correlated to Obsessive-Compulsive Symptoms. J Nerv Ment Dis.

[66] Li R, Kajanoja J, Lindblom J, Korja R, Karlsson L, Karlsson H (2022). The role of alexithymia and perceived stress in mental health responses to COVID-19: A conditional process model [published correction appears in J Affect Disord. 2022 Apr 9;:]. J Affect Disord.

[67] Aaron RV, Blain SD, Snodgress MA, Park S (2020). Quadratic Relationship Between Alexithymia and Interoceptive Accuracy, and Results From a Pilot Mindfulness Intervention. Front Psychiatry.

[68] Katsifaraki M, Tucker P (2013). Alexithymia and burnout in nursing students. J Nurs Educ.

[69] Cameron K, Ogrodniczuk J, Hadjipavlou G (2014). Changes in alexithymia following psychological intervention: a review. Harv Rev Psychiatry.

[70] Hill AP, Curran T (2016). Multidimensional Perfectionism and Burnout: A Meta-Analysis. Pers Soc Psychol Rev.

[71] Lin G-X, Szczygieł D (2022). Perfectionistic parents are burnt out by hiding emotions from their children, but this effect is attenuated by emotional intelligence. Personality Individ Differ.

[72] Li Z. A study on the relationship between adult attachment, coping style and psychological stress. Harbin Engineering University,2018.

[73] Han W, Pan H, Li S, Wei C, Chen J (2023). Current status and influencing factors of parenting burnout in children with autism spectrum disorders. Nurs Res.

[74] Ardic A (2020). Relationship between parental burnout level and perceived social support levels of parents of children with autism spectrum disorder. Int J Educ Methodology.

[75] Gillis A, Roskam L (2020). Regulation between daily exhaustion and support in parenting: A dyadic perspective. Int J Behav Dev.

[76] Arrindell WA (2003). Culture's consequences: Comparing values, behaviors, institutions, and organizations across nations. Behav Res Ther.

[77] Lewis C, Lamb ME (2003). Fathers’ influences on children’s development: The evidence from two-parent families. Eur J Psychol Educ.

