# Supplementary material for: A systematic review of parental burnout and related factors among parents
Source: BMC Public Health. 2024 Feb 5;24:376. doi: 10.1186/s12889-024-17829-y (PMC10840230; doi:10.1186/s12889-024-17829-y)
Supplement: Supplementary file 1 — Additional file 1: Table S1. Searching strategies. Table S2. Quality appraisal of the reviewed studies. [file 12889_2024_17829_MOESM1_ESM.docx]

**Supplementary files**

| Table S1: searching strategies | | |
| --- | --- | --- |
| Pubmed | ((parenting [MeSH Terms]) AND (burnout, psychological [MeSH Terms] OR Psychological Burnout OR Burn-out Syndrome OR Burn out Syndrome OR Burnout OR Burnout Syndrome OR Burn-out OR Burn out OR Psychological Burn-out OR Burn-out, Psychological OR Psychological Burn out) )OR( (parental [MeSH Terms]) AND (burnout, psychological [MeSH Terms] OR Psychological Burnout OR Burn-out Syndrome OR Burn out Syndrome OR Burnout OR Burnout Syndrome OR Burn-out OR Burn out OR Psychological Burn-out OR Burn-out, Psychological OR Psychological Burn out)) OR Burnout Parental | 239 |
| Web of science | ((TS=(parenting AND ( burnout, psychological OR Psychological Burnout OR Burn-out Syndrome OR Burn out Syndrome OR Burnout OR Burnout Syndrome OR Burn-out OR Burn out OR Psychological Burn-out OR Burn-out, Psychological OR Psychological Burn out))) OR TS=( parental AND (burnout, psychological OR Psychological Burnout OR Burn-out Syndrome OR Burn out Syndrome OR Burnout OR Burnout Syndrome OR Burn-out OR Burn out OR Psychological Burn-out OR Burn-out, Psychological OR Psychological Burn out))) OR TS=(Burnout Parental) | 809 |
| EBSCO | (parenting AND (burnout, psychological OR Psychological Burnout OR Burn-out Syndrome OR Burn out Syndrome OR Burnout OR Burnout Syndrome OR Burn-out OR Burn out OR Psychological Burn-out OR Burn-out, Psychological OR Psychological Burn out) )OR( parental AND (burnout, psychological OR Psychological Burnout OR Burn-out Syndrome OR Burn out Syndrome OR Burnout OR Burnout Syndrome OR Burn-out OR Burn out OR Psychological Burn-out OR Burn-out, Psychological OR Psychological Burn out)) OR Burnout Parental | 423 |
| CNKI | Parenting burnout/parental burnout | 169 |
| WanFang | Parenting burnout/parental burnout | 30 |

| Table S2. Quality appraisal of the reviewed studies. | | | | | | | | | | | | | | | | | | |
| --- | --- | --- | --- | --- | --- | --- | --- | --- | --- | --- | --- | --- | --- | --- | --- | --- | --- | --- |
| Author & Year | (1) | (2) | (3) | (4) | (5) | (6) | (7) | (8) | (9) | (10) | (11) | (12) | (13) | (14) | (15) | (16) | Sum of score | Score% |
| Zhou et al.(2023) | 3 | 1 | 2 | 0 | 2 | 0 | 2 | 2 | 2 | 1 | NA | 3 | 2 | NA | 2 | 0 | 22/42 | 52.4% |
| Zhanget al.(2023) | 0 | 2 | 3 | 1 | 2 | 1 | 2 | 2 | 2 | 2 | NA | 3 | 2 | NA | 1 | 0 | 23/42 | 54.8% |
| Li et al.(2023) | 2 | 2 | 3 | 1 | 2 | 1 | 2 | 1 | 2 | 1 | NA | 0 | 0 | NA | 1 | 1 | 19/42 | 45.2% |
| [Zhuo](https://pubmed.ncbi.nlm.nih.gov/?term=Zhuo R[Author]) et al.(2023) | 1 | 3 | 3 | 1 | 2 | 1 | 2 | 2 | 2 | 2 | NA | 3 | 2 | NA | 1 | 3 | 28/42 | 66.7% |
| [Zach](https://pubmed.ncbi.nlm.nih.gov/?term=Gerber Z[Author])et al.(2021) | 1 | 2 | 3 | 2 | 2 | 2 | 2 | 1 | 2 | 1 | NA | 3 | 2 | NA | 1 | 3 | 27/42 | 64.3% |
| [Lin](https://pubmed.ncbi.nlm.nih.gov/?sort=fauth&term=Lin+Y&cauthor_id=37359855) et al.(2023) | 2 | 2 | 3 | 2 | 2 | 1 | 2 | 1 | 2 | 2 | NA | 3 | 2 | NA | 2 | 3 | 29/42 | 69% |
| [Huang](https://pubmed.ncbi.nlm.nih.gov/?sort=fauth&term=Huang+Y&cauthor_id=37286938)et al.(2023) | 1 | 2 | 2 | 2 | 2 | 2 | 2 | 1 | 2 | 1 | NA | 3 | 2 | NA | 1 | 3 | 26/42 | 61.9% |
| [Lin](https://pubmed.ncbi.nlm.nih.gov/?sort=fauth&term=Lin+GX&cauthor_id=36046000) et al.( 2022) | 0 | 3 | 3 | 1 | 2 | 1 | 2 | 1 | 2 | 1 | NA | 3 | 2 | NA | 2 | 3 | 26/42 | 61.9% |
| [Sodi e](https://pubmed.ncbi.nlm.nih.gov/?sort=fauth&term=Sodi+T&cauthor_id=33206468)t al.(2020) | 0 | 2 | 2 | 1 | 2 | 1 | 2 | 2 | 2 | 1 | NA | 2 | 2 | NA | 1 | 2 | 22/42 | 52.4% |
| [Kawamoto](https://pubmed.ncbi.nlm.nih.gov/?sort=fauth&term=Kawamoto+T&cauthor_id=29973893) et al. (2018) | 1 | 3 | 3 | 2 | 2 | 1 | 2 | 1 | 2 | 2 | NA | 3 | 3 | NA | 2 | 2 | 29/42 | 69% |
| [Szczygieł](https://pubmed.ncbi.nlm.nih.gov/?sort=fauth&term=Szczygie%C5%82+D&cauthor_id=33201567) et al. (2020) | 0 | 3 | 2 | 2 | 3 | 1 | 2 | 1 | 2 | 1 | NA | 3 | 2 | NA | 1 | 3 | 26/42 | 61.9% |
| [Liu](https://pubmed.ncbi.nlm.nih.gov/?sort=fauth&term=Liu+S&cauthor_id=36637590) et al.(2023) | 0 | 2 | 3 | 2 | 3 | 1 | 2 | 1 | 2 | 2 | NA | 3 | 2 | NA | 2 | 2 | 27/42 | 64.3% |
| [Sekułowicz](https://pubmed.ncbi.nlm.nih.gov/?sort=fauth&term=Seku%C5%82owicz+M&cauthor_id=35162210) et al. (2022) | 0 | 2 | 3 | 2 | 2 | 2 | 2 | 2 | 2 | 1 | NA | 3 | 2 | NA | 0 | 2 | 25/42 | 59.5% |
| [Séjourné](https://pubmed.ncbi.nlm.nih.gov/?sort=fauth&term=S%C3%A9journ%C3%A9+N&cauthor_id=29517340) et al. (2018) | 0 | 3 | 3 | 1 | 2 | 1 | 2 | 1 | 2 | 1 | NA | 3 | 2 | NA | 0 | 3 | 24/42 | 57.1% |
| [Roskam](https://pubmed.ncbi.nlm.nih.gov/?sort=fauth&term=Roskam+I&cauthor_id=33758826) et al. (2021) | 0 | 3 | 3 | 2 | 2 | 2 | 2 | 2 | 2 | 1 | NA | 3 | 2 | NA | 1 | 3 | 28/42 | 66.7% |
| [Prandstetter](https://pubmed.ncbi.nlm.nih.gov/?sort=fauth&term=Prandstetter+K&cauthor_id=35068912) et al. (2023) | 3 | 3 | 3 | 1 | 2 | 1 | 2 | 1 | 2 | 2 | NA | 3 | 1 | NA | 0 | 3 | 27/42 | 64.3% |
| [Konrad e](https://pubmed.ncbi.nlm.nih.gov/?term=Piotrowski K[Author])t al. (2023) | 2 | 3 | 3 | 2 | 3 | 2 | 3 | 2 | 3 | 1 | NA | 3 | 3 | NA | 2 | 3 | 35/42 | 83.3% |
| Hong et al. (2022) | 1 | 3 | 2 | 1 | 2 | 2 | 2 | 1 | 2 | 2 | NA | 3 | 1 | NA | 2 | 3 | 27/42 | 64.3% |
| Favez et al.(2023) | 3 | 2 | 3 | 2 | 2 | 2 | 2 | 2 | 2 | 1 | NA | 3 | 2 | NA | 2 | 3 | 31/42 | 73.8% |
| Gannagé et al. (2020) | 0 | 2 | 3 | 2 | 2 | 1 | 1 | 1 | 2 | 1 | NA | 3 | 2 | NA | 1 | 3 | 24/42 | 57.1% |
| [Meeussen](https://pubmed.ncbi.nlm.nih.gov/?sort=fauth&term=Meeussen+L&cauthor_id=30455656) et al. (2018) | 1 | 3 | 3 | 2 | 2 | 3 | 2 | 3 | 2 | 2 | NA | 3 | 2 | NA | 2 | 3 | 33/42 | 78.6% |
| [Lindström](https://pubmed.ncbi.nlm.nih.gov/?sort=fauth&term=Lindstr%C3%B6m+C&cauthor_id=21414025) et al. (2011) | 1 | 2 | 2 | 2 | 3 | 3 | 2 | 2 | 2 | 2 | NA | 3 | 2 | NA | 2 | 2 | 30/42 | 71.4% |
| [Lin](https://bpspsychub.onlinelibrary.wiley.com/authored-by/Lin/Gao%E2%80%90Xian) et al.(2022) | 0 | 3 | 3 | 2 | 2 | 1 | 1 | 0 | 1 | 1 | NA | 3 | 3 | NA | 2 | 3 | 25/42 | 59.5% |
| [Vigouroux](https://pubmed.ncbi.nlm.nih.gov/?term=Vigouroux SL[Author]) et al. (2018) | 0 | 3 | 2 | 2 | 2 | 0 | 0 | 0 | 1 | 1 | NA | 3 | 2 | NA | 1 | 0 | 17/42 | 40.5% |
| [Meryem](https://pubmed.ncbi.nlm.nih.gov/?sort=fauth&term=K%C3%BCt%C3%BCk+M%C3%96&cauthor_id=33459915) et al. ( 2021) | 1 | 3 | 3 | 2 | 2 | 2 | 2 | 1 | 2 | 2 | NA | 3 | 2 | NA | 2 | 3 | 30/42 | 71.4% |
| Cheng et al.(2021) | 0 | 3 | 1 | 0 | 2 | 2 | 1 | 1 | 1 | 0 | NA | 3 | 2 | NA | 1 | 2 | 19/42 | 45.2% |

(1) explicit theoretical framework; (2) statement of aims/objectives in main report; (3) clear description of research setting; (4) evidence of sample size considered in terms of analysis; (5) representative sample of target group of a reasonable size; (6) description of procedure for data collection; (7) rationale for choice of data collection tool(s); (8) detailed recruitment data; (9) statistical assessment of reliability and validity of measurement tool(s) (quantitative studies only); (10) fit between research question and method of data collection (quantitative studies only); (11) fit between research question and format and content of data collection tool (qualitative studies only); (12) fit between research question and method of analysis (quantitative studies only); (13) good justification for analytic method selected; (14) assessment of reliability of analytic process (qualitative studies only); (15) evidence of user involvement in design; (16) strengths and limitations critically discussed. NA:Not available
